# N-(2-Hydroxyphenyl)-2-propylpentanamide Modulates HDAC1 and GPER1 Expression in a Rodent Model of Triple-Negative Breast Cancer

**DOI:** 10.3390/biomedicines14020322

**Published:** 2026-01-30

**Authors:** Cynthia Ramírez-Farías, Javier Ventura-Juárez, Argelia Calvillo-Robledo, Manuel Enrique Ávila-Blanco, Daniel González-Blas, José Correa-Basurto, Andrés Quintanar Stephano

**Affiliations:** 1 Departamento de Fisiología y Farmacología, Universidad Autónoma de Aguascalientes, Aguascalientes 20131, Mexico; cynthia.ramirez.farias@gmail.com (C.R.-F.); argelia.calvillo@edu.uaa.mx (A.C.-R.); 2 Departamento de Morfología, Universidad Autónoma de Aguascalientes, Aguascalientes 20131, Mexico; jventur@correo.uaa.mx (J.V.-J.); meab21@gmail.com (M.E.Á.-B.); 3 Laboratorio de Anatomía Patológica, Hospital General ISSSTE, Avenida Universidad 410, San Cayetano, Aguascalientes 20010, Mexico; dagob73@hotmail.com; 4 Laboratorio de Diseño y Desarrollo de Nuevos Fármacos e Innovación Biotecnológica, Escuela Superior de Medicina, Instituto Politécnico Nacional, Ciudad de México 11340, Mexico; corrjose@gmail.com

**Keywords:** TNBC, iHDAC1, GPER1, drug resistance, tumor fibrosis, CAFs, tumoral microenvironment

## Abstract

**Background:** Triple-negative breast cancer (TNBC) is one of the most aggressive breast cancer subtypes due to its rapid growth, poor prognosis, and low response to chemotherapies owing to a lack of therapeutic targets and drug resistance. Histone deacetylases (HDACs) induce stromal changes that increase extracellular matrix density through the activity of cancer-associated fibroblasts (CAFs). HDACs are overexpressed in TNBC and have been linked to the activation and sustained activity of CAFs. Additionally, HDAC inhibitors decrease the fibroblastic activity. **Objectives:** We aimed to analyze the antifibrotic effect of the N-(2′-hydroxyphenyl)-2-propylpentanamide (HO-AAVPA), an inhibitor of the HDAC1, 6, and 8 (iHDAC) on TNBC. **Methods:** The TNBC (4T1) cell line was inoculated under the dorsal skin in mice to develop a TNBC tumor. CAF’s activation was determined by measuring collagen-1 and alpha-smooth muscle actin (α-SMA), as well as their association with the G-protein-coupled estrogenic receptor (GPER1) and HDAC1 expression. **Results:** Dose of 20 mg/kg of HO-AAVPA decreased tumor fibrosis by inducing decreased collagen-1 and alpha-smooth muscle actin (α-SMA) levels and increased GPER1 expression. Moreover, HO-AAVPA reduced the activation and activity of CAFs. **Conclusion:** Our results support the notion that HDAC1 inhibition may be a novel approach to sensitizing resistant tumor cells to chemotherapy and radiotherapy by increasing GPER1 expression, and thus the use of antiproliferative GPER1 agonists/antagonists, at least in the early stages, without causing significant changes in liver function or morphological alterations.

## 1. Introduction

Breast cancer (BC) is a heterogeneous and complex disease in which several internal and external factors impact its development and progression [1]. With an estimated 2.29 million new cases diagnosed and over 666,000 deaths per year, BC is the most diagnosed cancer worldwide and the leading cause of cancer-related death among women [2]. Current therapeutic strategies show limited efficacy in patients with relapsed or metastatic cancer due to the development of resistance to treatments, which was formerly attributed to genetic factors [3,4]. However, a wide variety of direct and indirect mechanisms contribute to cancer resistance to drugs and radiation, including genetic and epigenetic modifications, metabolic alterations, tumor cell diversity, increased DNA repair, and changes in the tumor stroma [5,6]. Therefore, the search for new pharmacological alternatives for the control or cure of BC continues to be of great social and health importance.

Since the 1960s, rapid, non-genomic responses to estrogen have been observed in ovariectomized rats, involving the production of cAMP and calcium mobilization [7,8]. These effects, initially attributed solely to the estrogen receptor (ER)-α, were later linked to the G protein-coupled receptor (GPCR) GP estrogen receptor 1 (GPER) [9,10], which mediates rapid estrogen signaling, including ERK activation [11] and c-Fos expression, independently of ERα [12]. Revankar et al. (2005) identified GPER as a distinct intracellular estrogen receptor whose localization modulates its functional activity. The subsequent development of selective ligands (G-1, G-15, and G-36) enabled precise investigation of GPER-mediated pathways. In triple-negative breast cancer (TNBC)**,** GPER has garnered increasing attention due to its association with tumor progression and specific clinicopathological features. Ongoing studies aim to elucidate its role in estrogen signaling and its potential contribution to TNBC development and therapy resistance [13].

Revankar et al. (2005) established GPER as a distinct estrogen receptor with intracellular localization influencing its activity. The development of selective ligands (G-1, G-15, G-36) has enabled the targeted study of GPER. In the context of triple-negative breast cancer (TNBC), GPER has gained attention for its association with tumor progression and clinicopathological features. Ongoing research focuses on elucidating its role in estrogen signaling and its implications in TNBC development and therapy resistance [13].

The breast stroma is a crucial tissue component of the mammary gland, comprising fibroblasts, adipocytes, neuronal and immune cells, and protein components of the extracellular matrix (ECM), including proteoglycans, hyaluronic acid, fibronectin, collagen, laminin, growth factors, cytokines, chemokines, metabolites, and antibodies. All these components are known collectively as the tumor microenvironment (TME) [14,15,16,17]. A strong relationship between the stroma density and tumor development/progression has been established, with a 2- to 6-fold higher BC susceptibility in women with increased stroma density, which is primarily correlated with an increase in collagen deposits, high insulin-like growth factor 1 (IGF-1) and metalloproteinase 3 inhibitor (TIMP-3) levels [18,19].

The ECM serves as a scaffold for tumor development and contributes to the cell migration [20], metabolic changes [21] and drug resistance in tumor cells [22], mainly due to the increase in matrix protein deposits and other functions regulated by cancer-associated fibroblasts (CAFs). There are 81 genes related to treatment resistance, 11 of which are involved in ECM formation, including the gene that codes for collagen 1 (COL1A1), which provides tamoxifen resistance [23] and radiotherapy resistance [24]. The increase in matrix components creates a rigid and close physical barrier of proteins that hinders the entry of chemotherapeutics due to the capture of drugs without affecting the diffusion of oxygen and nutrients [21], and the delivery of metabolites, as these are small and polar molecules [16,17]. In vitro comparative studies using monocultures and co-cultures of breast carcinoma cells and BC-associated fibroblasts (BCAFs) have revealed that only the BCAFs increase in growth factors, interleukins (IL-6 and 8), and matrix metalloproteinases (MMP2 and MMP11), all of which are associated with increased tumor growth, angiogenesis, metastasis, and invasiveness [25]. Tumor cells secrete Platelet-Derived Growth Factor (PDGF) and Transforming Growth Factor-β (TGF-β) to stimulate the transformation of normal fibroblasts (NFs) into BCAFs, which in turn secrete growth factors that promote tumor cell proliferation and drug resistance [26].

TGF-β participates in the formation of an aberrant cell that activates fibroblasts and modulates the TME, increasing ECM deposition and the formation of a physical barrier (e.g., cells, proteins, carbohydrates, and lipids), thereby hindering the entrance of antitumor drugs. This, in turn, contributes to the epithelial–mesenchymal transition (EMT) and cell proliferation [27,28,29]. It is known that the deletion or downregulation of histone deacetylase 1 (HDAC1) mRNA suppresses TGF-β1-induced EMT and cell migration [30].

NFs are quiescent cells that regulate various processes, such as wound healing, tissue inflammation, and fibrosis, and are associated with anti-tumorigenic activity [31,32]. Although the mechanism by which NFs transform to CAFs has not been fully elucidated, this process is not due to mutations in the NF genome but rather changes in histone acetylation, methylation, phosphorylation, ubiquitination, ADP ribosylation, SUMOylation and DNA methylation, which can lead to fibroblast reprogramming, allowing them to acquire pro-tumorigenic phenotypes, secondary to modifications in their transcriptional activity, which leads to TME and remodeling [33,34,35]. The primary post-translational modifications mediated by HDACs (histone deacetylation) can lead to gene inactivation, subsequently downregulating processes that are conducive to tumor cell initiation and progression, in which CAFs play a critical role [36]. The development of compounds that inhibit HDACs could create new therapeutic strategies, either as independent drugs or adjuvants, which could reverse the CAFs phenotype into NFs; in other words, changing the pro-tumoral activity of the CAFs into NFs cells, i.e., restoring the microenvironment of the BC to make it less pro-tumoral, and thus favoring that tumor cells be more sensitive to antitumoral treatments [37,38,39,40].

Some pan-HDAC inhibitors (HDACi), such as valproic acid (VPA) and suberoylanilide hydroxamic acid (SAHA), are effective against some types of cancer. However, they have low specificity for HDAC isoforms. They are associated with severe side effects, such as hepatotoxicity and hyperammonemia with VPA [41] and myelosuppression and thrombocytopenia with SAHA [42].

**Figure 1:**
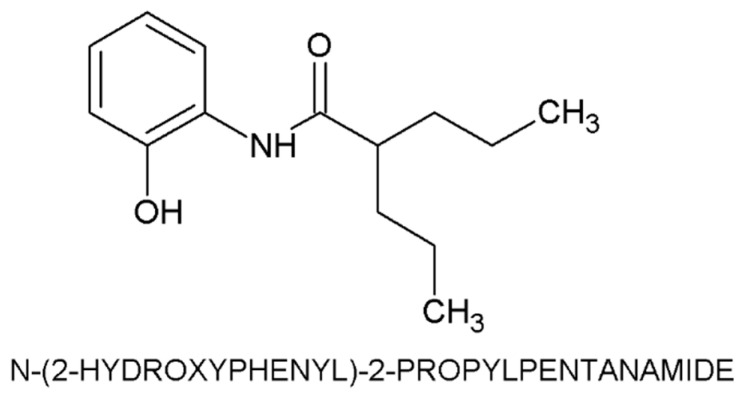
Chemical structure of the compound N-(2-hydroxyphenyl)-2-propylpentanamide. Adapted from Prestegui-Martel et al. J Enzyme Inhib Med Chem, 2016 [50].

The high mortality linked to chemotherapeutic resistance, which is driven by increased intratumoral fibrosis and the side effects of HDACi, highlights the need for new compounds that offer antiproliferative benefits with fewer adverse effects. The N-(2′-hydroxyphenyl)-2-propylpentanamide HO-AAVPA) [43,44], VPA-derived compound, which in vitro studies have demonstrated that it has antiproliferative effects on TNBC, and in vivo, it exhibits less adverse effects (Figure 1) [45,46,47]. The antiproliferative effects of the HO-AAVPA may have been caused by the HDAC inhibition influence in the cell death (apoptosis) [48] and EMT mechanism from CAFs into NF [49]. If this is true, it is essential to evaluate the antifibrotic effects of HO-AAVPA under in vivo conditions. Thus, the present work aimed to analyze the anti-HDAC1 effects of HO-AAVPA on MET, through the evaluation of CAFs activation and permanent activity and the possible reversion of intratumoral fibrosis in a murine model of TNBC (4T1). Liver histopathology changes were also evaluated to assess the hepatotoxic effects of HO-AAVPA.

## 2. Materials and Methods

### 2.1. Synthesis of the Compound N-(2-Hydroxyphenyl)-2-propylpentanamide (HO-AAVPA)

The synthesis of HO-AAVPA was carried out according to the synthetic route outlined in our work group’s report [50]. Briefly, the synthesis of HO-AAVPA proceeds by first activating valproic acid (VPA) (Sigma-Aldrich, Merck KGaA, Darmstadt, Germany) through its conversion to the reactive intermediate 2-propylpentanoyl chloride using oxalyl chloride (Sigma-Aldrich, Merck KGaA, Darmstadt, Germany) at 0 °C, followed by an amide coupling reaction where this acyl chloride is treated with ortho-hydroxy-aniline (Sigma Sigma-Aldrich, Merck KGaA, Darmstadt, Germany) and subsequently quenched with a base like sodium bicarbonate (Sigma-Aldrich, Merck KGaA, Darmstadt, Germany) to yield the final derivative.

### 2.2. Animals

Female mice aged 9–12 weeks from the Balb-C strain were provided by the Vivarium of the Autonomous University of Aguascalientes. All mice were kept on a 12:12 h light-dark cycle with constant ambient temperature (21 ± 1 °C) and humidity (50 ± 7%). Food (Lab Diet 5010, Purina, St. Louis, MO, USA) and water were available ad libitum. All experiments were carried out in accordance with the ethical principles and regulations specified by NOM-062-ZOO-199 (México), and reviewed and approved by the Institutional Bioethics Committee, called CEADI-UAA (Ethics Committee for the Use of Animals in Teaching and Research at the Autonomous University of Aguascalientes) (file number CEADI-UAA/05/2025).

#### Experimental Groups and Randomization

To establish the breast cancer model, 22 female mice were first inoculated subcutaneously with 4T1 cells. After one week, once palpable tumor development was confirmed, animals were randomly allocated into the tumor control (n = 10) and tumor + vehicle (n = 10) groups. During the 22-day observation and follow-up period, the mortality rate associated with tumor progression was recorded. Following completion of this initial phase (21 days), an additional 30 animals were inoculated with 4T1 cells to evaluate the pharmacological treatments. One week after tumor establishment, animals were randomly assigned into the following treatment groups: VPA 600 mg/kg (n = 7), HO-AAVPA 20 mg/kg (n = 7), HO-AAVPA 40 mg/kg (n = 7), and HO-AAVPA 80 mg/kg (n = 7). All treatments were administered once daily for 21 consecutive days.

### 2.3. Breast Cancer Induction

The 4T1 murine mammary carcinoma cell line was obtained from the American Type Culture Collection (ATCC, Cat. No. CRL-2539™, Manassas, VA, USA) and maintained according to ATCC recommendations on a Roswell Park Memorial Institute (RPMI 1640, GIBCO^TM^) (Thermo Fisher Scientific, Cat. 11875-093, Waltham, MA, USA) medium enriched with 10% fetal bovine serum (FBS) from GIBCO™ (Thermo Fisher Scientific, Cat. No. 16000044, Waltham, MA, USA). Once the confluence was reached (70–80%), cells were harvested using EDTA (cat. E8008, Sigma-Aldrich, Merck KGaA, Darmstadt, Germany). Cells were then centrifuged at 2500 rpm and resuspended in RPMI 1640 with 10% FBS. Cells were counted using the Neubauer chamber technique [51] and Trypan blue (cat. 93595-100ML, Sigma-Aldrich, Merck KGaA, Darmstadt, Germany) stain. A suspension of 7.5 × 10^5^ cells/50 μL was subcutaneously injected into the interscapular space of each animal [52].

### 2.4. Drug Treatments

**Figure 2:**
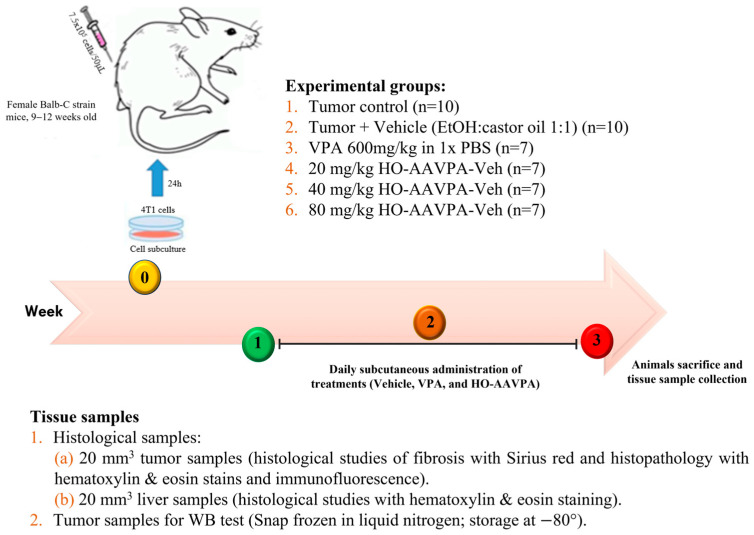
Experimental design showing treatment groups and sample collection for histopathology, immunohistochemistry, and Western blot analyses. “n” indicates the number of induced animals per group.

Different treatments were prepared: Sodium valproate (cat. P4543-10G, Sigma-Aldrich, Merck KGaA, Darmstadt, Germany) in PBS 1x (Cat. P3813-10PAK, Sigma-Aldrich, Merck KGaA, Darmstadt, Germany) (600 mg/kg body weight/20 mL), vehicle 1:1 mixture of ethanol (cat. 02870, Sigma-Aldrich, Merck KGaA, Darmstadt, Germany) and castor oil (cat. C5135, Sigma-Aldrich, Merck KGaA, Darmstadt, Germany) (20 μL), HO-AAVPA in a 1:1 mixture of ethanol and castor oil (20, 40 and 80 mg/kg body weight/20 mL). Subcutaneous injections were administered in ventral area daily for 21 days (Figure 2). At the end of the treatment period, surviving animals in each group were euthanized by overdose of sodium pentobarbital (150 mg/kg). The final number of animals included in the subsequent histological, immunohistochemical, and biochemical analyses was as follows: tumor control (n = 8), tumor + vehicle (n = 9), VPA 600 mg/kg (n = 5), HO-AAVPA 20 mg/kg (n = 7), HO-AAVPA 40 mg/kg (n = 7), and HO-AAVPA 80 mg/kg (n = 7).

### 2.5. Histology

Four weeks after 4T1 cell injection, animals were euthanized, and liver and tumor tissues were collected for histological evaluation. Samples were fixed in 15–17% neutral-buffered formalin, dehydrated through a graded ethanol series, cleared in xylene, and embedded in paraffin. Paraffin blocks were sectioned at 5 µm thickness, and liver and tumor sections were stained with hematoxylin (cat. 51275, Sigma-Aldrich, St. Louis, MO, USA) and eosin (cat. 318906, Sigma-Aldrich, St. Louis, MO, USA) following standard protocols [53]. Liver sections were additionally stained with periodic acid–Schiff (PAS) reagent (cat. 101646, Sigma-Aldrich, St. Louis, MO, USA) [54] to evaluate glycogen content. Tumor histological grade was determined using the Bloom–Richardson (SBR) system [55]. Sirius Red staining (cat. 365548, Sigma-Aldrich St. Louis, MO, USA) was performed on tumor sections to assess intratumoral fibrosis [56]. Histopathological evaluation and image acquisition were performed using optical microscopy with digital microphotography, and quantitative analyses were conducted with Image Pro Plus v11 (Media Cybernetics, Rockville, MD, USA). A portion of tumor tissue was snap-frozen in liquid nitrogen and stored at –80 °C for subsequent Western blot analysis.

#### 2.5.1. Cell Death Area Evaluation

Cell death was quantified in H&E-stained tumor sections by analyzing seven microscopic fields per tissue sample. The scale bar was used to calibrate the pixel-to-micrometer ratio, and the total tissue area (mm^2^) within each field was measured. Regions exhibiting characteristic features of cell death (pyknosis, karyorrhexis, or acellular zones) were delineated, and their area was quantified. The percentage of cell death was calculated as (cell death area/total tissue area) × 100 [57].

#### 2.5.2. Fibrosis Evaluation

Fibrosis quantification was performed using Sirius Red staining as previously described [58]. Nine microscopic fields per sample were analyzed. Images were converted to grayscale and thresholded to isolate collagen-positive regions. Fibrosis was expressed as the ratio of thresholded collagen area to total tissue area within each field, following the methodology described by Vogel. et al. (2015) and Schindelin et al. (2012) [57,59].

### 2.6. Immunofluorescence

Immunofluorescence was performed on tumor sections as described by Joshi and Yu (2017) [60] using primary antibodies against HDAC1 (cat. GTX100513, dilution 1:500, from GeneTex Inc., Irvine, CA, USA) and α-SMA (cat. GTX100034, 1:700 dilution, from GeneTex Inc., Irvine, CA, USA). Anti-rabbit Alexa 488 fluorochrome-conjugated secondary antibodies (cat. A11008, 1:500 dilution, Invitrogen™, Thermo-Fisher Scientific Inc., Waltham, MA, USA) were used for HDAC1 labeling. In contrast, rhodamine labeling (cat. AP132R, 1:500 dilution, Millipore™, Merck KGaA, Darmstadt, Germany) was used for α-SMA. Samples were fixed with 2% paraformaldehyde (PFA) (Cat. P6148-500MG, Sigma-Aldrich, Merck KGaA, Darmstadt, Germany) between each immunostaining. Nuclear staining was performed with Hoechst 33342 for 10 min (cat. 14530, 1:1000 dilution, Sigma-Aldrich, Merck KGaA, Darmstadt, Germany).

#### Quantification of HDAC1- and α-SMA-Positive Cells

ImageJ Pro v11 (Media Cybernetics, Rockville, MD, USA) was used to quantify HDAC1- and α-SMA-positive cells. Fifteen microscopic fields per tissue sample were analyzed. The software was first calibrated using the scale bar located in the lower right corner of each image to establish a reference pixel-to-micrometer ratio and to determine the corresponding field area in micrometers squared (µm^2^), which was subsequently converted to millimeters squared (mm^2^). A grid overlay was applied to facilitate systematic cell counting, and the Cell Counter tool was used to identify and count positively stained cells, displaying green fluorescence for HDAC1 and red fluorescence for α-SMA. The number of positive cells per mm^2^ was then calculated by normalizing the counted cells to the corresponding field area [57].

### 2.7. Western Blot

#### 2.7.1. Protein Extraction

Protein extraction was performed from tumor tissue snap-frozen in liquid nitrogen and stored at −80 °C. Samples were lysed on ice using freshly prepared RIPA buffer consisting of 50 mM Tris-HCl (cat. T3253-250G, Sigma-Aldrich, Merck KGaA, Darmstadt, Germany), 150 mM NaCl (cat. 3624-01; JT Baker, Phillipsburg, NJ, USA), 1% NP-40 (cat. 85124, Thermo Fisher Scientific, Waltham, MA, USA), 0.5% sodium deoxycholate (cat. SRE0046, Sigma-Aldrich, Merck KGaA, Darmstadt, Germany), and 0.1% SDS (cat. L3253-250G, Sigma-Aldrich, Merck KGaA, Darmstadt, Germany) (https://www.abcam.com/protocols/general-western-blot-protocol, accessed on 20 Mach 2024). The buffer was supplemented with a protease inhibitor cocktail (Mini cOmplete™, cat. 11836153000, Roche, Merck KGaA, Darmstadt, Germany). Lysates were incubated on ice for 30 min with occasional gentle vortexing, followed by centrifugation at 14,000× *g* for 15 min at 4 °C. The supernatants were collected for protein quantification.

#### 2.7.2. Protein Quantification

Protein concentration was determined using the Pierce BCA assay (cat. 23225, Thermo Fisher Scientific, Waltham, MA, USA) according to the manufacturer’s instructions.

#### 2.7.3. Immunoblotting

GPER1 samples were denatured at room temperature to prevent aggregation, while GAPDH and COL1 samples were boiled at 95 °C for 5 min. Electrophoresis was performed by loading 40 μg of total protein per lane into a 7% SDS-polyacrylamide gel. Proteins were transferred onto Immun-Blot^®^ PVDF membranes (0.2 μm pore size; Bio-Rad Laboratories, Hercules, CA, USA; cat. 1620177). Membranes were blocked for 2 h at room temperature with 5% non-fat milk (Svelty^®^, Nestlé, Mexico) prepared in Tris-buffered saline (TBS; 20 mM Tris-base, Promega, Madison, WI, USA; Cat. H5131; 137 mM NaCl, JT Baker, Phillipsburg, NJ, USA; Cat. 3624-01). After blocking, membranes were incubated overnight at 4 °C with primary antibodies against COL1A1 (cat. GTX112731, 1:1500, GeneTex Inc., Irvine, CA, USA), GPR30 (cat. GTX107748, 1:500, GeneTex Inc., Irvine, CA, USA), and GAPDH (cat. G9545, 1:10,000, Sigma-Aldrich, Merck KGaA, Darmstadt, Germany).

Following primary antibody incubation, membranes were washed three times (10 min each) with TBS containing 0.05% Tween-20 (Promega, Madison, WI, USA; cat. H5152), and subsequently incubated for 2 h at room temperature with HRP-conjugated anti-rabbit secondary antibody (cat. A0545, 1:2000, Sigma-Aldrich, Merck KGaA, Darmstadt, Germany). Protein bands were developed using the Immobilon™ chemiluminescent substrate kit (cat. WBKLS0500, Millipore, Merck KGaA, Darmstadt, Germany) and visualized using the imaging system described in Section 2.8. Densitometric quantification was performed using Image Lab™ software (version 6.1.0, Bio-Rad Laboratories Inc., Hercules, CA, USA). Relative expression levels were calculated according to Pillai et al. (2020) [61] by normalizing all target protein signals to GAPDH.

### 2.8. Instruments

The following instruments were used throughout the experimental procedures. Tissue embedding was performed using a paraffin embedding station (Leica EG1150H, Leica Microsystems, Wetzlar, Germany), and tissue sectioning was carried out with a rotary microtome (Leica RM212RT, Leica Microsystems, Wetzlar, Germany). Histological staining was conducted using an automated slide stainer (Leica ST4040, Leica Microsystems, Wetzlar, Germany). Optical analyses were performed using an Axioscop 40 optical microscope (Zeiss™, Carl Zeiss AG, Oberkochen, Germany), and microphotographs of liver and tumor tissue sections were acquired using a CoolSNAP-Pro color digital camera coupled to the microscope, with image capture managed through Image-Pro Plus software v11 (Media Cybernetics, Rockville, MD, USA). Protein electrophoresis for Western blot assays was conducted using a Mini-PROTEAN system (Bio-Rad, Hercules, CA, USA), and chemiluminescent signal detection was performed with a DNR Bio-Imaging System (Microchem 4.2, Jerusalem, Israel).

### 2.9. Statistical Analysis

A one-way analysis of variance (one-way ANOVA) was performed for the relative expression of GPER1 and COL1 (Western blot), followed by Tukey’s tests. A normality test was performed on the cell death area, the number of cells positive for HDAC1 and α-SMA, and fibrosis inhibition, which revealed that the data were non-parametric. Therefore, the Kruskal–Wallis test and its corresponding Dunn test were performed. Statistical tests were performed using the GraphPad Prism program version 8 (GraphPad, San Diego, CA, USA). Data are expressed as means ± S.D. *p*-values less than 0.05 were considered significant.

## 3. Results

### 3.1. Effects of VPA and HO-AAVPA on Tumor Histology

**Figure 3:**
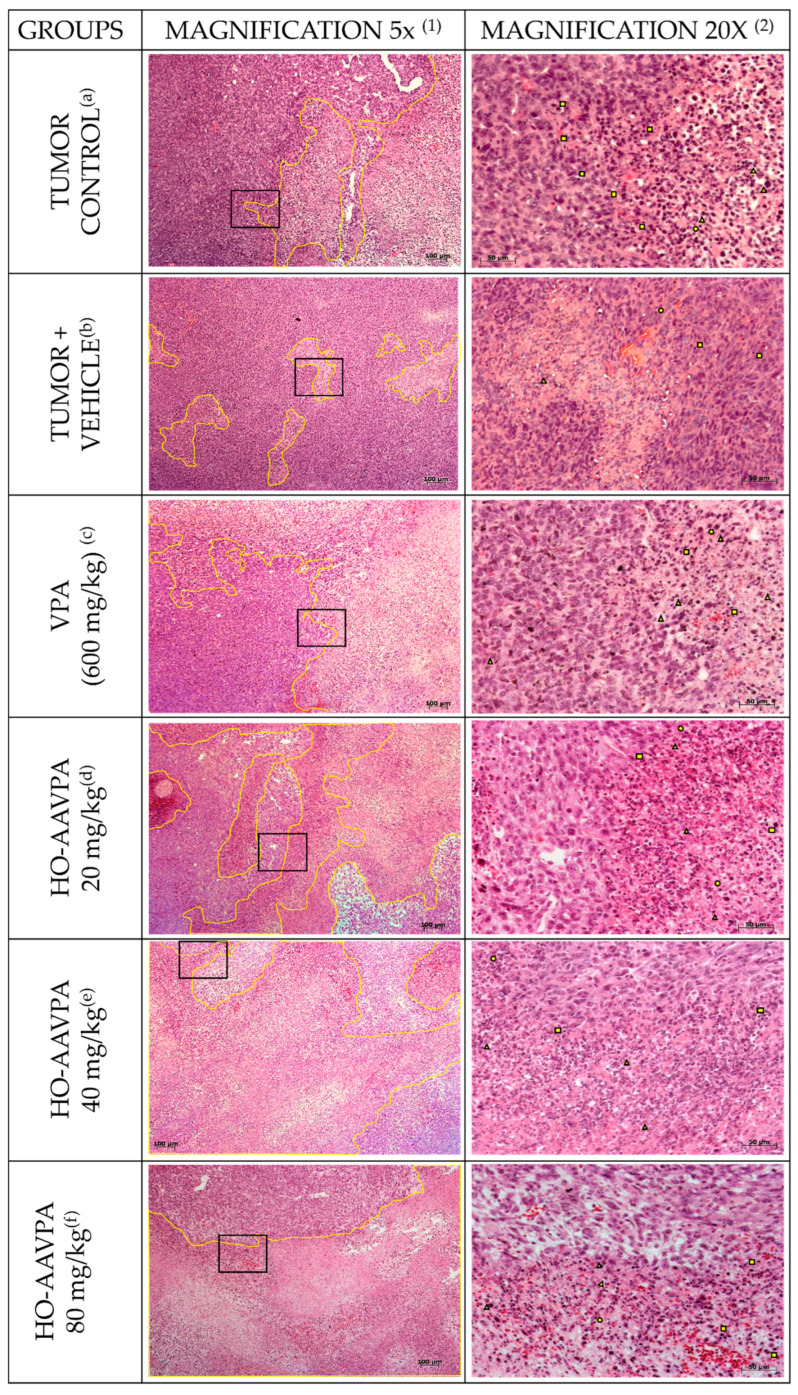
Stromal and morphological alterations induced by HO-AAVPA. (**a**) Tumor control group; (**b**) tumor + vehicle group; (**c**) VPA (600 mg/kg) group; (**d**) HO-AAVPA 20 mg/kg group; (**e**) HO-AAVPA 40 mg/kg group; and (**f**) HO-AAVPA 80 mg/kg group. Panels are arranged in two columns: column 1 shows areas of cell death (yellow line) surrounded by viable cells; column 2 shows morphological changes, including pyknotic nuclei (squares), karyolysis (triangle), and karyorrhexis (circle). Hematoxylin and eosin staining was used. Images were captured at 5× and 20× magnification.

**Figure 4:**
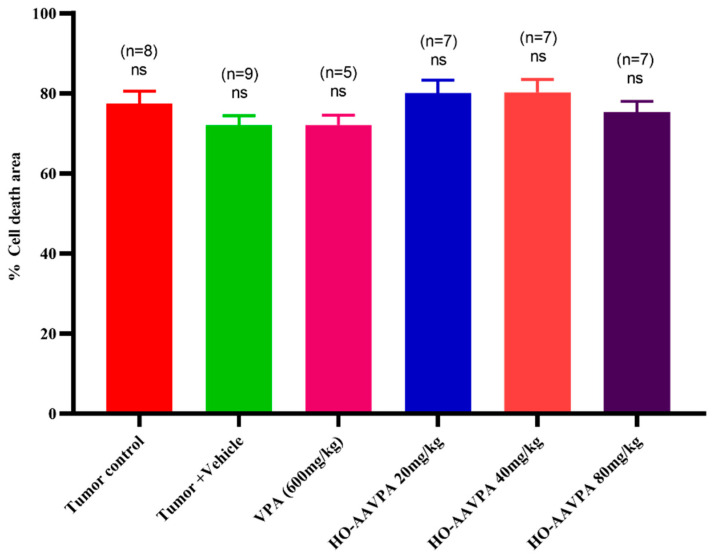
Effect of HO-AAVPA on cell death. No statistically significant differences were observed among experimental groups. Data are presented as mean ± standard deviation (SD). Statistical analysis was performed using the Kruskal–Wallis test.

The tumoral tissues of all experimental groups exhibited similar changes in cell death, accompanied by stromal changes, such as increased zones of cellular death surrounded by viable tumor cells (Figure 3a–f). Theses death zones displayed nuclear pyknosis, karyorrhexis, karyolysis, cytoplasmic vacuolization, and large acellular areas (Figure 3a–f). Despite these histological changes, the size of the necrotic areas not differ significantly in size between the experimental groups (Figure 4).

#### Hepatic Morphology Changes in Response to VPA and HO-AAVPA

**Figure 5:**
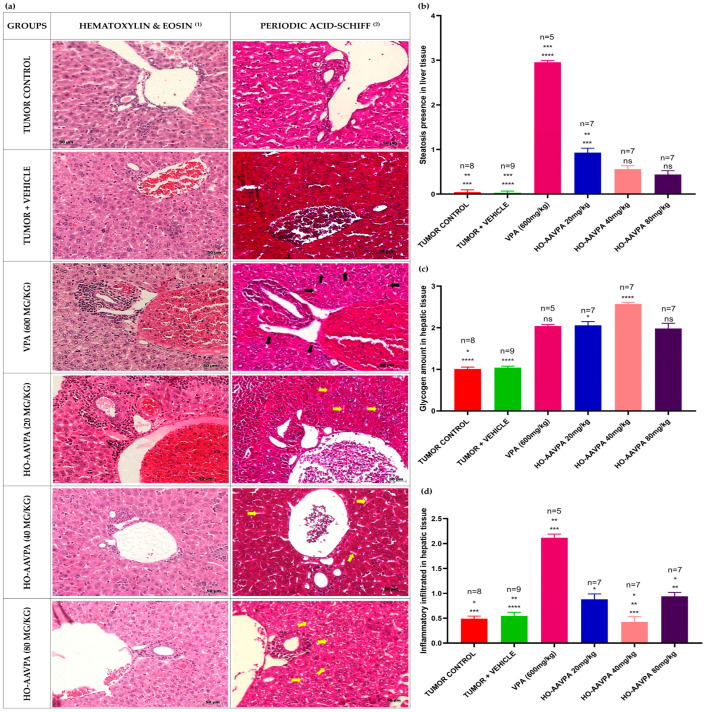
Morphological changes in liver tissue. (**a**) Representative micrographs showing hepatocyte alterations: column (1) corresponds to hematoxylin and eosin (H&E) staining, showing the general morphology of hepatic tissue; column (2) corresponds to periodic acid–Schiff (PAS) staining, where micro- and macrovesicular steatosis (black arrows) are observed in the VPA-treated group and glycogen deposits (yellow arrows) in the HO-AAVPA 20, 40, and 80 mg/kg groups, evidenced by spaces between the cell membrane and the nucleus. (**b**) Significant differences were observed in the degree of steatosis, with an increase in the VPA and HO-AAVPA 20 mg/kg groups compared with the control, whereas the HO-AAVPA 40 and 80 mg/kg groups exhibited a marked reduction in steatosis compared with the VPA and HO-AAVPA 20 mg/kg groups. (**c**) Quantification of glycogen vesicles in liver tissue in response to the treatments. A significant increase was detected in the HO-AAVPA 20 mg/kg and 40 mg/kg groups compared with the tumor control, as well as a significant difference between the tumor + vehicle group and the HO-AAVPA 40 mg/kg group. (**d**) Differences in inflammatory infiltrate. A significant increase was found in the VPA 600 mg/kg group compared with the tumor control, tumor + vehicle, and HO-AAVPA 40 mg/kg groups; in the HO-AAVPA 80 mg/kg group compared with the tumor control and HO-AAVPA 40 mg/kg groups; and in the HO-AAVPA 20 mg/kg group compared with the HO-AAVPA 40 mg/kg group. Statistical analysis was performed using the Kruskal–Wallis test followed by Dunn’s post hoc test. **** *p* < 0.0001, *** *p* < 0.001, ** *p* < 0.01, * *p* < 0.05. “n” indicates the number of animals evaluated per group.

Table 1 presents the evaluation of morphological changes in hepatocytes, with VPA inducing macro- and microvesicular steatosis in 60–90% of the liver tissue, as well as moderate glycogen accumulation and mixed inflammatory infiltrates (lymphocytes and polymorphonuclear cells) around the portal triad, consistent with a NASH-type lesion. HO-AAVPA-treated groups induced from very-low to low steatosis, moderate to high glycogen deposits and non-significant inflammatory infiltrates. Control groups did not show steatosis nor inflammatory infiltrate, whereas glycogen deposits were normal. These results were supported by H&E and periodic acid-Schiff (PAS) staining (Figure 5a).

Histological analysis of liver tissue revealed distinct morphological changes among the experimental groups (Figure 5a). In the VPA-treated group, hepatocytes exhibited micro- and macrovesicular steatosis, characterized by the presence of cytoplasmic lipid vacuoles (indicated by black arrows). In contrast, animals treated with HO-AAVPA at doses of 20, 40, and 80 mg/kg showed the presence of glycogen deposits (yellow arrows), evidenced by spaces between the plasma membrane and the nucleus. Significant differences were observed in the degree of steatosis (Figure 5b). The VPA and HO-AAVPA 20 mg/kg groups displayed a marked increase in lipid accumulation compared with the control group. In contrast, HO-AAVPA at 40 and 80 mg/kg significantly reduced the extent of steatosis relative to both the VPA and HO-AAVPA 20 mg/kg groups. Quantification of glycogen vesicles (Figure 5c) showed a significant increase in the HO-AAVPA 20 and 40 mg/kg groups compared with the tumor control. Moreover, a significant difference was detected between the tumor + vehicle and HO-AAVPA 40 mg/kg groups. Analysis of inflammatory infiltrate (Figure 5d) revealed a substantial increase in the VPA 600 mg/kg group compared with the tumor control, tumor + vehicle, and HO-AAVPA 40 mg/kg groups. Similarly, a higher inflammatory response was observed in the HO-AAVPA 80 mg/kg group compared with the tumor control and HO-AAVPA 40 mg/kg groups, as well as between the HO-AAVPA 20 mg/kg and 40 mg/kg groups.

### 3.2. Changes in Tumoral Stroma Fibrosis by VPA and HO-AAVPA

#### Sirius Red Stain

**Figure 6:**
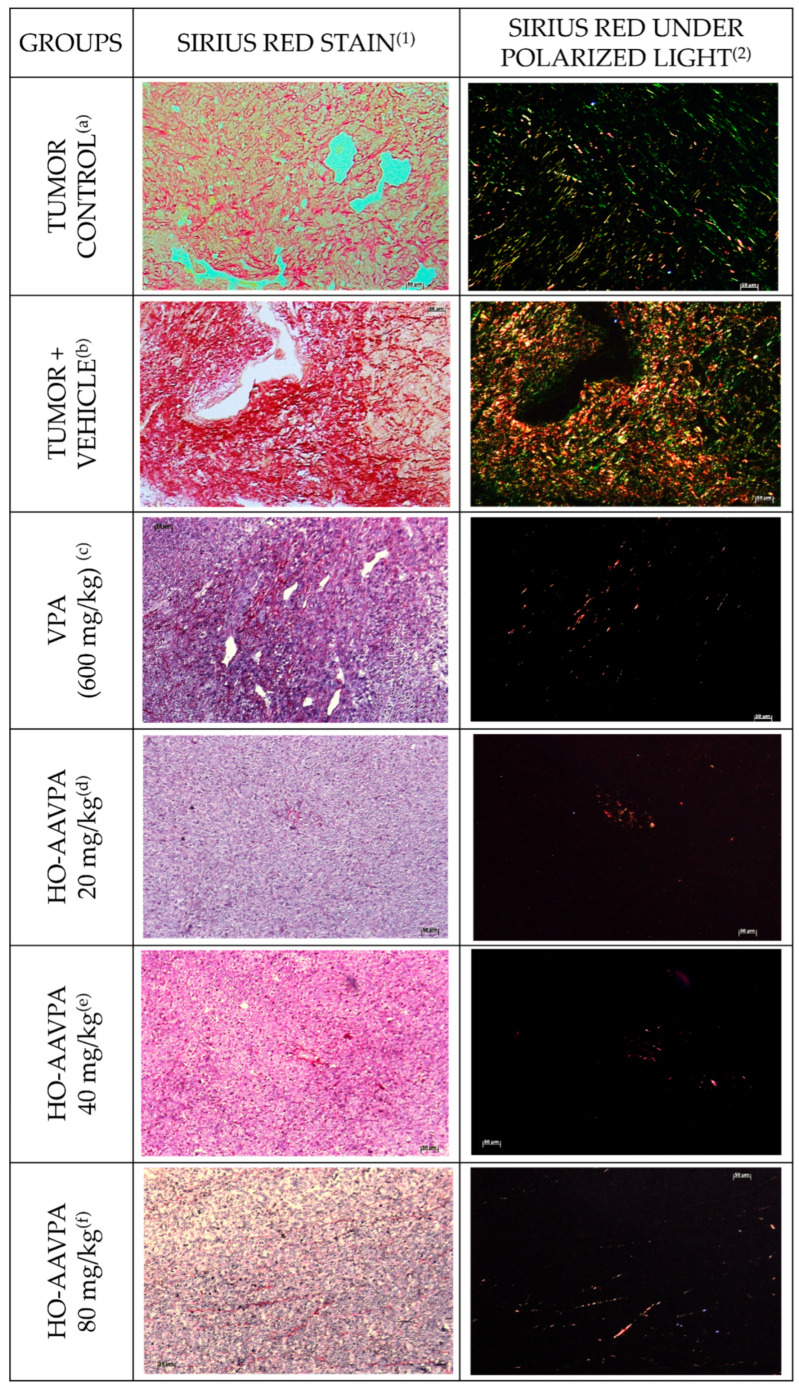
Sirius Red staining and collagen visualization under polarized light in tumor tissue from treated and control groups. Representative micrographs of Sirius Red–stained sections showing fibrotic areas in tumor tissues from the control, tumor + vehicle, VPA (600 mg/kg), and HO-AAVPA-treated groups (20, 40, and 80 mg/kg). Column (1) corresponds to Sirius Red staining observed under bright-field illumination, highlighting collagen distribution within tumor tissue, whereas column (2) shows the same sections observed under polarized light, allowing differentiation of collagen fiber types, where red fibers correspond to collagen type I and green fibers to collagen type III. (**a**,**b**) The tumor control and tumor + vehicle groups exhibit extensive and dispersed collagen fiber clusters with intermediate thickness. (**c**–**f**) Treatment with VPA and HO-AAVPA significantly reduced the fibrotic area, exhibiting fewer and thinner collagen fibers compared with the control groups. Images illustrate a dose-dependent decrease in collagen I and III deposition in animals treated with HO-AAVPA.

Sirius Red staining revealed a marker reduction in fibrotic area in animals treated with VPA and HO-AAVPA (20, 40 and 80 mg/kg) (Figure 6c–f). In these groups, a noticeably lower number of red-stained fibers was observed in the tissue compared with both the tumor control group (Figure 6a) and the tumor + vehicle groups (Figure 6b). Under polarized light, Sirius Red staining allowed for the visualization of collagen deposits as birefringent, “fluorescent-like” fibers. In the tumor control and tumor + vehicle groups, large and dispersed clusters of collagen fibers with intermediate thickness were evident, with red fibers corresponding to collagen type I and green fibers to collagen type III on a dark background (Figure 6a,b). In contrast, animals treated with VPA (600 mg/kg) and HO-AAVPA (20, 40 and 80 mg/kg) sowed a pronounced reduction in both collagen I and III deposition, with only sparse and thin fiber bundles remaining (Figure 6c–f).

**Figure 7:**
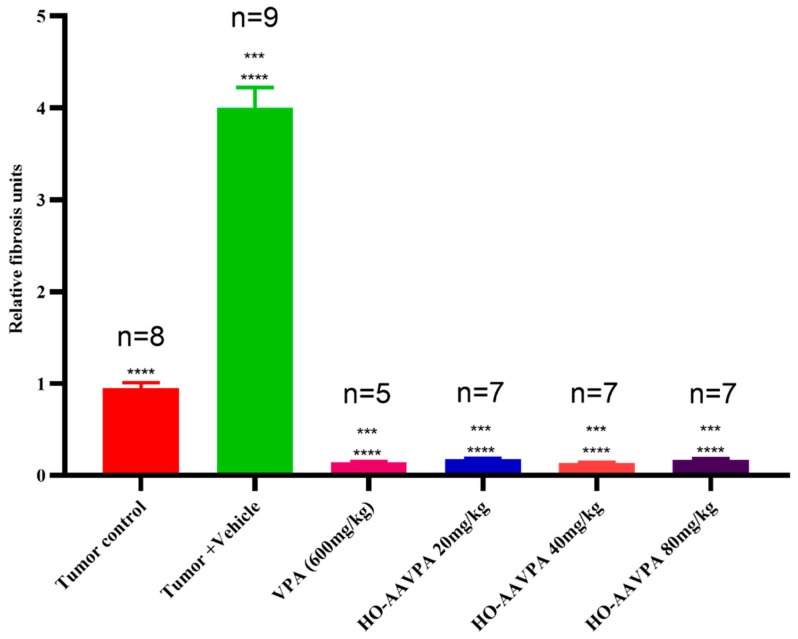
Quantification of fibrosis in experimental groups (relative units). A significant reduction in fibrosis was observed in the VPA and HO-AAVPA treatment groups compared with the tumor control group (**** *p* < 0.0001; *** *p* < 0.001). Additionally, all treatments (VPA and HO-AAVPA at 20, 40, and 80 mg/kg) showed significantly lower fibrosis levels compared with the tumor + vehicle group. HO-AAVPA at 20 and 40 mg/kg reduced fibrosis by 8.76- and 8.79-fold, respectively, compared to tumor control. Meanwhile, VPA and HO-AAVPA (20–80 mg/kg) showed reductions of 9.58, 9.65, 9.66, and 9.31-fold, respectively, compared to tumor + vehicle. Statistical analysis was performed using the Kruskal–Wallis test followed by Dunn’s post hoc test. “n” represents the number of animals evaluated.

HO-AAVPA significantly decreases collagen stores (relative units), decreasing by 95.1%, 95.3%, 93.5% and 93.2% in the VPA, 20, 40, and 80 mg/kg HO-AAVPA-treated groups, respectively, as compared with the tumor + vehicle group. Likewise, in comparison to the tumor group, HO-AAVPA reduced the stores of collagen by 80%, 80.6%, 99.9% and 72.2%, respectively. All together, these findings demonstrate the antifibrotic potential of HO-AAVPA in comparison to the high production of fibrosis induced by the vehicle (Figure 7).

### 3.3. HDAC1 and α-SMA Expression in Tumor Tissue with VPA and HO-AAVPA

**Figure 8:**
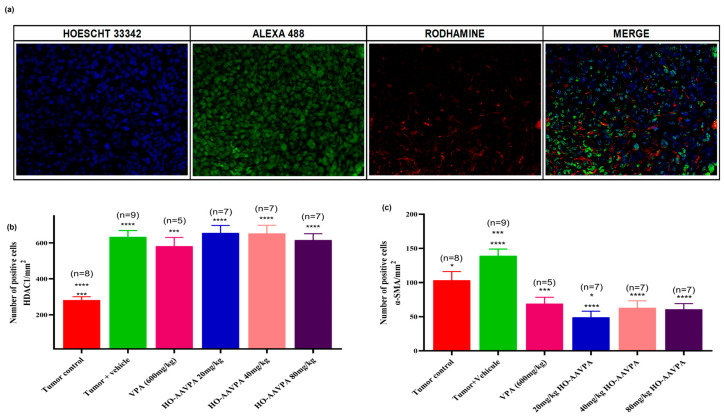
Immunofluorescence analysis of HDAC1 and α-SMA expression in tumor tissue. (**a**) Nuclei stained with Hoechst 33342; HDAC1 detected with anti-HDAC1-Alexa 488 (green), α-SMA with anti-αSMA rhodamine (red). Images captured at 20× magnification. (**b**) Quantification of HDAC1-positive cells. (**c**) Quantification of α-SMA-positive cells. Data are presented as mean ± SD. Statistical analysis: Kruskal–Wallis with Dunn’s post hoc test. **** *p* < 0.0001, *** *p* < 0.0005, * *p* < 0.05. “n” represents of animals evaluated.

The immunofluorescence results show the differences in biomarker expression across the experimental groups. Hoechst staining was used for dot visualization of cell nuclei, Alexa 488 for HDAC1 expression (bright green dots), and rhodamine for α-SMA in the cytoplasm of activated cancer-associated fibroblasts. The three channels were then merged to provide a combined view (Figure 8a). The analysis revealed an increase in HDAC1 expression and a decrease in α-SMA expression among the different experimental groups. A significant increase in HDAC1-positive cells was detected in the tumor + vehicle, VPA, and HO-AAVPA groups (20, 40, and 80 mg/kg), with 2.3-, 2.2-, 2.4-, 2.3-, and 2.2-fold elevations, respectively, compared with the tumor control (Figure 8b). Conversely, VPA and HO-AAVPA treatments markedly reduced the proportion of CAFs expressing α-SMA (FACα-SMA+), showing 1.9, 2.7, 2.3, and 2.2-fold decreases relative to the tumor + vehicle group, and 1.5, 2.1, 1.8, and 1.7-fold decreases compared with the tumor control (Figure 8c).

### 3.4. Expression of COL1A1 and GPER1 with VPA and HO-AAVPA

**Figure 9:**
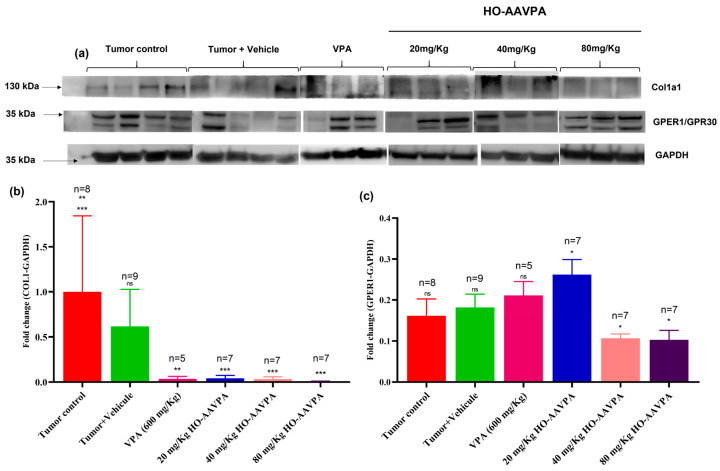
Expression of GPER1 was analyzed by Western blot in tumor tissue. (**a**) Representative blots for total lysates from different experimental groups using anti-GAPDH, anti-COL1, and anti-GPER1 antibodies. (**b**) Fold change in COL1 expression. (**c**) Fold change in GPER1 expression quantified with Image-Lab™ software. Data are presented as mean ± SD (minimum n = 5). Statistical analysis: one-way ANOVA with Tukey’s post hoc test. *** *p* < 0.001, ** *p* < 0.005, * *p* < 0.05.

The GPER1 plays a crucial role in tumor cell metastasis and invasiveness, as well as in the survival of BC patients [62]. We assessed the expression of COL1A1 and GPER1 in induced tumor tissues (Figure 9a). The Western blot results indicated that HDACi treatments significantly down-regulated COL1A1 expression (Figure 9b). Regarding GPER1, its expression was more significantly decreased following treatment with 40 and 80 mg/Kg HO-AAVPA than the 20 mg/Kg and 600 mg/Kg treatments, but not compared to the remaining groups (Figure 9c).

## 4. Discussion

Morphological changes in tumor cells and stroma can indicate tumor malignancy [63]. We used the SBR (maximum score = 9 points) to evaluate the tumor grades. Based on this scale, the tumor tissues had characteristics of a high-grade carcinoma. The SBR evaluates three characteristics: tubule formation (˂10%, 3 points), nuclear characteristics of pleomorphism (marked variation 3 points), and the mitotic index (>10 atypical mitoses in 10 fields 3 points) [55,64] (Figure 4). Treatment with VPA and HO-AAVPA led to stromal and cell changes in tumor tissues; however, the differences in the sizes of cells death zones and tumor growth among the different treatment doses were not significant (Figure 3). Although, the results indicated a slightly increase in cell death induced and a correlation between the dose and duration of treatment with respect to the antiproliferative and proapoptotic effects of HDACis. An in vitro study by Prestegui-Martel et al. (2016) reported concentration-dependent antiproliferative effect of OH-AAVPA in various BC cell lines [50]. The differences in results obtained from in vitro and in vivo studies could be attributed to the fact that in vitro, the OH-AAVPA immediately reaches its biological targets, whereas in an in vivo model, it must cross physiological barriers to reach the tumor zone and biological targets. Genetic, epigenetic, metabolic, and physiological variability play important roles in the response to OH-AAVPA in animal models.

HDACis are well known as anticancer; among these, vorinostat (SAHA), romidepsin (FK228), belinostat (PXD101), and panobinostat (LBH589) have been approved by the U.S. Food and Drug Administration (FDA) or the European Medicines Agency for the treatment of cutaneous T-cell lymphoma (CTCL), peripheral T-cell lymphoma (PTCL), and multiple myeloma (MM). Additionally, chidamide (CS055), approved by the China Food and Drug Administration (CFDA), has been used for the treatment of PTCL and advanced BC (metastatic or locally recurrent and inoperable) [65]. VPA is an interesting drug suggested as an anticancer [66]. However, secondary effects associated with its use have been reported, including hepatotoxicity. Liver toxicity is mediated by the generation of reactive oxygen species (ROS) resulting from the mitochondrial β-oxidation of VPA to 4-ene VPA and subsequently to 2,4-diene VPA [67,68], which depletes hepatic glutathione, leading to oxidative stress. Micro-vesicular steatosis has been observed in isolated hepatocytes and liver biopsies taken from animals who received 4-ene VPA [69]. In contrast, both macro- and micro-vesicular steatosis, particularly in the hepatolobular zone, have been found in epileptic patients who underwent prolonged VPA consumption [70]. In the present work, the VPA group exhibited micro-vesicular changes in hepatocytes (Figure 5) and increased animal mortality following two weeks of treatment confirming the hepatotoxic effects of VPA reported elsewhere [71]. The presence of vesicles observed in the hepatic tissue of animals from the VPA-treated group may plausibly be attributed to glycogen accumulation. This interpretation could be supported by findings from Oiso et al. (2011), who showed that HDAC1 inhibition decreases the expression of phosphoenolpyruvate carboxykinase (PEPCK), an enzyme responsible for the regulation of gluconeogenesis, and the transcription factor hepatocyte nuclear factor-4 alpha (HNF-4α), which participates in glucose and lipid metabolism [72,73]. PEPCK also down-regulates gluconeogenesis and indirectly promotes glycogenesis [72]. Although hepatic glycogen formation is characterized by vesicles, with some remnants of glycogen granules exhibiting rough edges in H&E stains, whereas lipid accumulation is characterized by smooth-edged vesicles as described by Hardisty and Brix [74].

The inhibitory effect of HO-AAVPA on HDAC1 [43,44] decreased the number of fibroblasts that expressed α-SMA. It is known that activated fibroblasts promote TME and tumor progression [75]. Kim and collaborators reported that Scriptaid, a class I HDACi, antagonizes TGFβ-mediated gene expression in CAFs. In an in vivo tumor model, it decreased ECM protein deposition, such as collagen I, which reduced its contractility and rigidity, thereby delaying tumor growth [75]. In cultured kidney epithelial cells, Trichostatin A (a class I and II HDACis) has been shown to suppress the EMT [76]. The decreased fibrosis by HO-AAVPA (Figure 6 and Figure 7) could be due to diminished CAF activity [77], thereby contributing to a potential reversal of the pro-tumor microenvironment through a decrease in the intrinsic mechanosensory processes of both CAFs and tumor cells, as well as their metastatic capacity [75].

The lightly diminishing antifibrotic effect seen in the highest dose of HO-AAVPA (80 mg/kg) may be explained by the induction of P-glycoprotein (P-gp) expression. P-gp is a protein that pulls out the anticancer drugs in HeLa tumor cells with HDAC1 inhibitors, as reported by Kim et al. (2009) and Wang et al. (2016) [78,79]. Thus, it is possible that there is a symbiotic relationship between tumor cells and CAFs (revised by C. Ramírez-Farías et al., 2021) [80], which promotes increased fibrosis, as was also observed in the present study (Figure 7). In support of this view, collagen deposits have been linked to tamoxifen resistance [23,80]. It is well known that genes related to ECM, such as COLA1A, are altered in advanced BC, a condition associated with high tumor cell invasiveness [23,81] and increased recruitment of stromal cells and ECM components, factors which provide a major metastatic phenotype [82]. Base on the present results, we can infer that decreased COL1A1 expression induced by HO-AAVPA (Figure 9b), could be used as a therapeutic strategy in patients that demonstrate resistance to first-line anticancer therapies due to high ECM density [75,76], in such cases, specific doses of HO-AAVPA (20–80 mg/kg) might sensitize tumor cells to anticancer treatments. This view is supported by the results of Chiu et al. (2013), who reported that in a TNBC in vivo model, using SAHA and radiation inhibited tumor growth and decreased pulmonary metastasis [83].

The participation of GPER1 in the development and progression of BC is not completely clear. However, its role as an inducer of cell proliferation and cell invasion in TNBC activation strongly suggests a good therapeutic target. Increased GPER1 expression promotes CAFs, both in tissue and isolated monocultures, favoring cell proliferation, migration, and phenotypic changes, resulting in a pro-oncogenic factor, including the EMT [84,85]. However, Chen et al. (2016) and Weißenborn et al. (2014) reported that GPER1 activation by G-1 (a GPER1 agonist) suppressed both BC cell proliferation and EMT in a concentration-dependent manner in TBNC cell line assays [86,87]. These observations support the view that increased GPER1 expression could be a promising therapeutic target for treating patients with TNBC, particularly in treatment-resistant tumors. In the present experiment, TNBC tumors treated with HO-AAVPA showed increased GPER1 protein expression in the 20 mg/kg and 600 mg/Kg groups and a subsequent decrease in expression in the 40 and 80 mg/kg groups (Figure 9c), which could be due to biphasic dose–response effects [88]. However, studies performed on immortalized endometrial cell lines (11z cells) treated with HDACis demonstrated decreased GPER1 expression, with ERs impacting cell proliferation and signal translation in a concentration-dependent manner [89]. Likewise, there are studies that show that HDACi downregulate GPER1 expression in in vitro experiments [48]. Still, its effect in EMT promotes the proliferation and modification has a beneficial role as suggested by De Francesco et al. (2018) [90]. However, it has been suggested that GPER1 overexpression is associated with pro-metastatic pathways [91].

Therefore, more studies are required to determine the role of GPER in EMT, as occurs in triple-negative cancer progression, and favoring restoration of breast cancer sensitivity to treatments.

## 5. Conclusions

We propose the use of HDAC inhibitors in the treatment of TNBC, based on their antifibrotic effect, as indicated by the decrease in CAFs and α-SMA in positive cells. The findings support the hypothesis that increased HDAC activity leads to a reduction in collagen deposition, without causing significant changes in liver function or morphological alterations, which suggests that HO-AAVPA does not possess toxic effects. Additionally, these results highlight the antiproliferative effect of GPER1 in modulating the course of TNBC during the early stages of the disease.

Further studies are necessary to determine the cellular, molecular, and epigenetic mechanisms in which HDAC inhibitors participate, as well as to clarify the dual role of GPER1 in the development and progression of this type of cancer.

## Figures and Tables

**Table 1:** Hepatic histopathological findings.

Experimental Group	Steatosis	Glycogen	Mixed Inflammatory Infiltrates
TUMOR CONTROL	−	+	+/−
TUMOR + VEHICLE	−	+	+/−
VPA 600 mg/Kg	+++ ^(#)^	++	++ ^(####)^
HO-AAVPA 20 mg/Kg	+	++	+
HO-AAVPA 40 mg/Kg	+/− ^(##)^	++/+++	+/−
HO-AAVPA 80 mg/Kg	+/− ^(###)^	++	+

(−) Negative, (+/−) Very low, (+) Low, (++) Moderate; (+++) High, (^#^) Macro and microsteatosis, (^##^) Isolated, (^###^) Microvesicular and focal, (^####^) NASH-type lesion (nonalcoholic steatohepatitis).

## Data Availability

The data supporting the findings of this study are available through drive, with the identifier [https://drive.google.com/drive/folders/15a0H18OagjkEaOS1cfeCMSfGHflMldZL?usp=drive_link] (accessed on 3 October 2025). The data include statistical analyses and immunofluorescence microphotographs. To access the data, please contact Cynthia Ramirez-Farias at cynthia.ramirez.farias@gmail.com.
